# Supplementary material for: Amoebae in Chronic, Polymicrobial Endodontic Infections Are Associated with Altered Microbial Communities of Increased Virulence
Source: J Clin Med. 2020 Nov 18;9(11):3700. doi: 10.3390/jcm9113700 (PMC7698792; doi:10.3390/jcm9113700)
Supplement: Supplementary file 1 [file jcm-09-03700-s001.pdf]

Journal of
Clinical Medicine

Supplementary Materials S1 Clinical details recorded, Sampling, DNA Extraction of Microbial DNA, 16S rRNA gene sequencing, Bioinformatic pipeline, Quantitative Polymerase Chain Reaction

Clinical details recorded

In addition to the microbial specimen, the following clinical features were also recorded for each patient: age, gender, infection type (primary or secondary, meaning initial or revision treatment), pain, tenderness to percussion, sinus tract and size of the periapical radiolucency, to determine the correlation between these features and microbial findings (Table 1). Prevalence of all clinical signs and symptoms (except periapical lesion size) were recorded on a binary scale [0 = absent, 1 = present], while the size of the radiolucency was measured in millimetres by two endodontic specialists on two-dimensional periapical radiographs (Planmeca Romexis, Coventry, UK).

Sampling

After anaesthesia, the tooth to be treated was isolated with a rubber dam (UnoDent, Essex, UK), and field decontamination was carried out before and after access opening, according to an established protocol, and shown to eliminate contaminating DNA (Data not shown). An access cavity was cut with a sterile bur under sterile saline irrigation (0.9% NaCl, Mölnlycke Health Care, Göteborg, Sweden), with contamination control samples taken. Root canal patency was assessed with a sterile K-file (Dentsply-Sirona, Ballaigues, Switzerland). For non-culture-based analysis, clinical samples were collected by inserting two paper points size 15 (Dentsply Sirona, USA) into the root canal. Each paper point was retained in the canal for 1 min with careful agitation, then was transferred to −80ºC storage immediately before further analysis. Cases of secondary endodontic treatment were sampled using the same protocol, with the exception that specimens were collected after removal of the coronal gutta-percha with Gates Glidden drills (Dentsply-Sirona, Switzerland). In this process, the apical gutta-percha point was retrieved by K-files without using chemical solvents except for sterile saline. In the cases of multi-rooted teeth in both primary and secondary endodontic treatment, the sample was obtained from the canal associated with the most extensive periapical lesion.

DNA Extraction of Microbial DNA

DNA was extracted from clinical samples of both primary and secondary endodontic infections by using a commercial DNA extraction kit (Sigma GenElute bacterial DNA extraction kit, Sigma Aldrich, St. Louis, MO, USA), following the manufacturer's instruction with modifications. After incubation in 2x10E5 units of lysozyme per reaction, addition of a 1.5 mm tungsten Carbide bead and DNA-free sand (Qiagen, Venlo, Netherlands), the cells were further disrupted using a FastPrep bead beater (MpBio, Santa Ana, CA, USA) at 6 m/s for 2 × 30 s. For all DNA extraction procedures, positive (*Enterococcus faecalis OMGS 3202)* and negative extraction controls, non-template and negative technical controls were included. DNA extracts from clinical samples and controls were resuspended in 100 µl Tris-EDTA buffer (pH 8.6), quantified and were stored at −20°C before use. For samples subjected to 16S Next-Generation sequencing, an unbiased amplification and clean-up of the DNA was carried out using Multiple Displacement Amplification (MDA, REPLI-g Mini Kit, Qiagen) using the manufacturer's protocol. Positive amplification was confirmed by 1% horizontal agarose gel electrophoresis.

16. S rRNA gene sequencing

The extracted DNA of 25 collected clinical specimens were analysed at using Illumina HiSeq Next generation sequencing was carried out at the Public Health England Genomics laboratory (Colindale, London, UK). 16S rRNA gene sequencing and samples were prepared for 16S ribosomal

RNA gene amplicons according to the Illumina protocol manual (Illumina, 2013). The hypervariable regions was amplified by using 16S Amplicon PCR with 600 cycles Forward Primer = 5′ CCTACG GGNGGCWGCAG 16S Amplicon PCR Reverse Primer = 5′ GACTACHVGGGTATCTAATCC [41].

Bioinformatic pipeline

Processing and taxonomic analysis of the sequence reads with matches to the GreenGenes (version 16) database was performed within dada2 using default parameters. Manually searched, non-aligned sequences were assigned by the NCBI Taxonomy (1,266,115 individual taxa) using the Lowest Common Ancestor (LCA) and the following thresholds: minimum bit-score: 80; max expectation value: 1.0×10^{-6}; top percentage of hits considered: 10%; minimum taxon support based on all assigned reads: 0.01%. Relative abundances and extraction of species-specific binned reads were calculated.

Quantitative Polymerase Chain Reaction

All quantitative (Q)PCR analyses for bacteria, fungi and amoebae were performed in triplicate. All PCR reactions were carried out in a total volume of 25 μL in SYBR PCR Mastermix (Applied Biosystems, Warrington, UK). Total bacterial density was determined using a 466-bp conserved 16S ribosomal RNA gene fragment, as described previously [42], at a concentration of 100 nM each for primers EubF (5′-TCCTACGGGAGGCAGCAGT-3′) and EubR (5′-GGACTACCAGGGTATCTAAT CCTGTT-3′). The fungal universal primers ITS-1 (5′-TCCGTAGGTGAACCTGCGG-3′) and ITS2 (5′-GCATCGATGAAGAACGCAGC-3′) were used to amplify the ITS1 region. Quantitative PCR assays were carried out using a temperature profile of 50 °C for 2 min, 95 °C for 10 min, followed by 45 cycles at 95 °C for 15 s and 60 °C for 60 s. The primers used for Entamoeba and *E.gingivalis* were used as described elsewhere [40], EGHF Primer (5′-TACCATACAAGGAATAGCTTTGTGAATAA-3′) and EGHR (5′-ACAATTGTAAATTTGTTCTTTTTCT-3′) were used to target the small subunit ribosomal 18S region of *E.gingivalis.* After thermal cycling, positive amplification and specificity of the amplicon was ascertained using Horizontal 2% Agarose gel electrophoresis. All positive bands amplified were excised, subjected to sanger sequencing (Eurofins Genomics, Cologne, Germany) and analysed using the Chromas Pro Software suite (Technelysium, Brisbane, Australia).

Results

Correlations with metadata, clinical signs and symptoms

Cases with sinus tracts yielded the largest number of bacterial associations (Figures 3 & 4), namely *Haemophilus parahaemolyticus* (which was the only species solely associated with lesion size above 5 mm in the present study), *Porphyromonas endodontalis*, *Bilophila* sp., *Catonella* sp., *Gemella* sp., *Corynebacterium* sp., *Megasphaera sp.*, significantly (the sole genus found to be associated with pain), *Mogibacteriaceae*, *Porphyromonas endodontalis*, *Prevotella melaninogenica*, *Prevotella pallens*, *Streptococcus anginosus* and *Treponema* sp.

Positive associations of reads and radiolucency size were observed for the OTU *Haemophilus parahaemolyticus* (p = 0.035), which was not observed in radiolucencies < 5mm. The OTU Pseudoramibacter/*Eubacterium sp.* was strongly correlated with lesions smaller than 5 mm (p = 0.021).

The association of age yielded significant associations for Coriobacteriaceae. (p = 0.028), *Prevotella nigrescens* (p = 0.044), *Bifidobacterium sp.* (p = 0.037), *Atopobium* sp. (p = 0.018), and *Aggregatibacter segnis* (p = 0.036), *Staphylococcus sp.* (p = 0.039), with *Staphylococcus aureus* (p = 0.03), for the group above and below the mean age, respectively. Gender differences were observed for for *Bifidobacterium longum*, *Bacteroides sp.* (p = 0.035) and *Butyrivibrio sp.* (p = 0.044) enriched in females, and *Lactobacillus zeae* (p = 0.038) for male patients.

Phylogenetic correlations at lower phylogenetic levels

The most abundant OTU (OTU068), Streptococcus sp. (excluding *S. sobrinus* and *S. anginosus,* which generally did not correlate with other *Streptococcus OTUs* or their respective correlates) showed the strongest correlations with *Actinomyces sp.* (R = 0.57), *Rothia sp.* (0.42), and at a *similar correlation strength to R. aeria* (0.48), *R. dentocariosa* (0.39), *Scardovia sp.* (0.57), *Veillonella dispar* (0.58), *Aggregatibacter segnis* (0.458) and *Atopobium sp.* (0.359). *A negative association was established for Actinotalea* (−0.264) and *Dialister sp.* (−0.219), *Alkalibacterium transvaalinensis* (−0.26), *Treponema soncranskii* (−0.240), *TG5* (−0.243), *Bacteroidales* (−0.203), *Oxalobacter* (−0.203) and *Propionibacterium acnes (−0.174).*

Species-level resolved *S. sobrinus* and *S. anginosus* did not typically follow these trends except for a correlation between *Streptococcus sp./S. sobrinus* and both *Veillonella dispar* (0.229 and 0.301, respectively) and *Cardiobacterium* sp. (0.357 and 0.497, respectively). An inverse correlation was observed with *Paracoccus sp.* and *Streptococcus sp.* (−0.261) and *S. anginosus* (0.447) and similarly for *Alkalimonas sp.* with −0.221 and 0.396 being the respective values obtained.

At species-unique level, a positive association of *S. anginosus* with *Megasphaera* sp. (0.98), *Campylobacter sp.* (0.98), *Actinotalea sp.* (0.64) and a negative correlation to *Atopobium* sp. (−0.115) was established. A further strong positive correlation to non-*P. acnes Propionibacterium sp.* (0.98) was established, the latter having a negative correlation to *S. sobrinus* (−0.16) but none to other *Streptococcus sp. members* (−0.03). *S. sobrinus* presented with a strong correlation to *Lactobacillus zeae* (0.67). *Mycoplasma sp.* displayed the strongest correlation with *Treponema sp.* (0.50), *Treponema amylovorum* (0.97), *Treponema socranskii* (0.62) and *Desulfovibrio sp* (0.85), suggesting common niches.

Association with epibionts

With regard to epibionts and bacterial parasites, *Candidatus Saccharibacterium* Group 3 (TM7-3), was shown to display a high species selectivity, with *Actinomyces odontolyticus* subsp. *actinosynbacter* XH001 described as the sole host to the oral clone TM7x [35], supported and potentially contributed to our data with a predominant, but specific correlation for genera within Actinomycetaceae and *Clostridiaceae,* as well as *Veilloneillaceae (Selenomonas sp.)*. For instance, the disease-specific oral clonotype I025 did not associate with *Actinomyces* sp., no association with signs or symptoms and group 3 representatives within this study could be ascertained, but with further insights into these elusive phyla, may be established for critical organisms associated with endodontic infections, and potential insights for co-culture studies established.

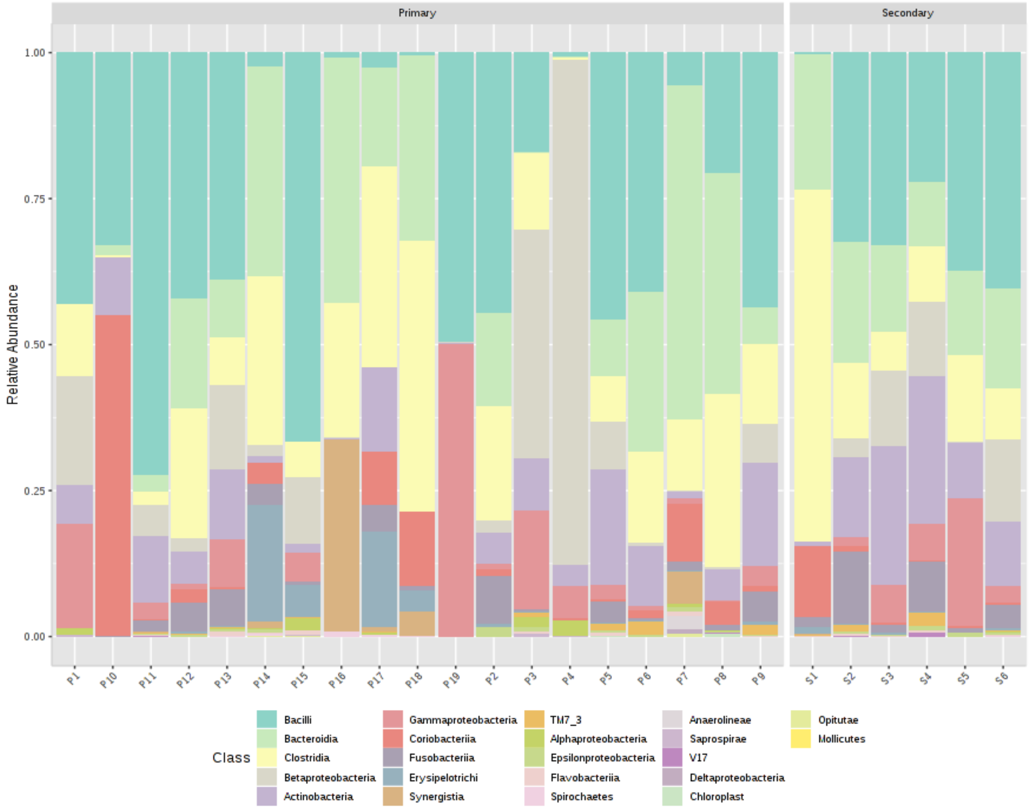

Supplementary Figure S1. Correlations across taxonomic levels and core microbiomes resolved to taxonomic levels. Correlations provided to class and treatment type (primary or secondary).

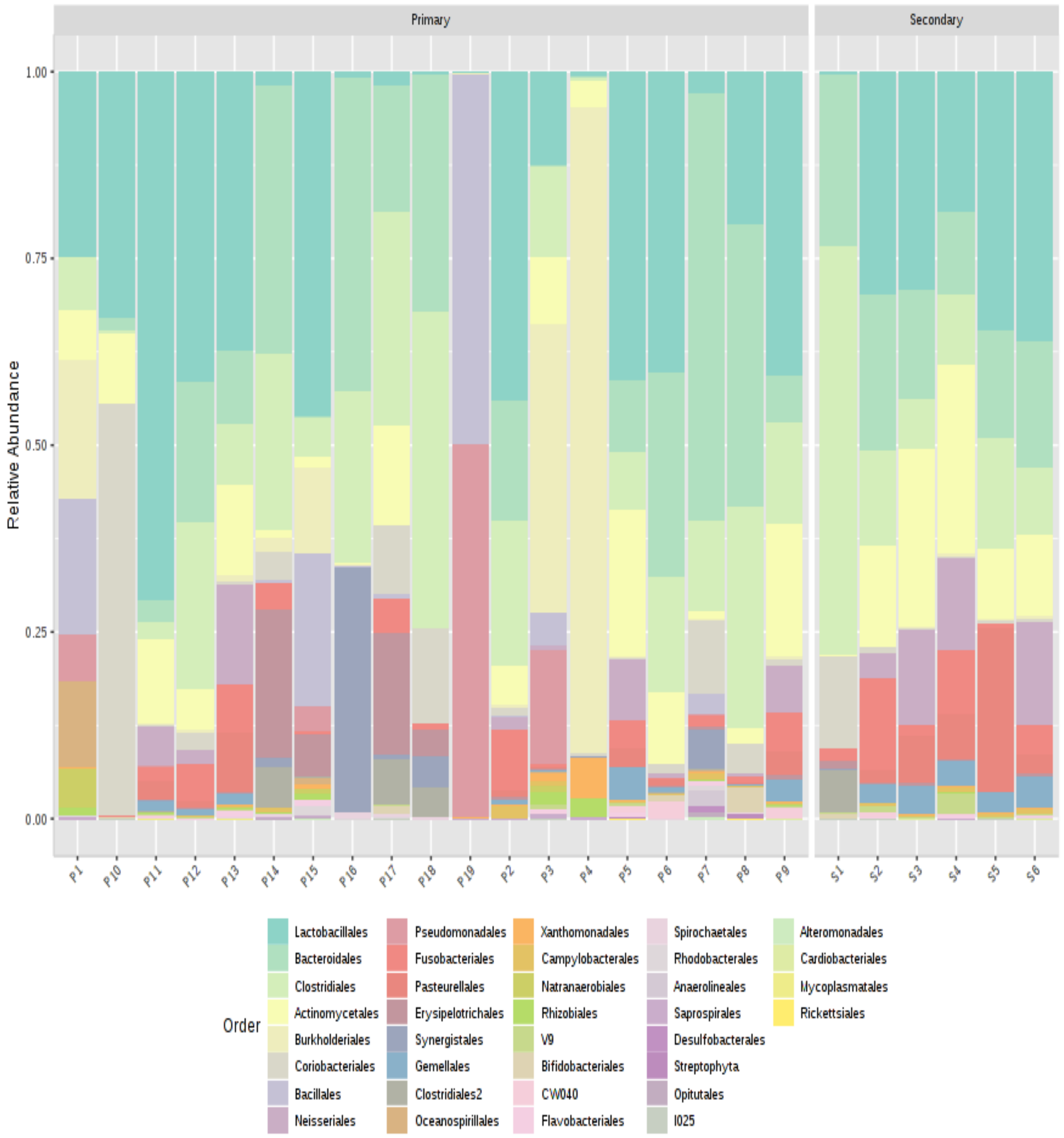

Supplementary Figure S2. Correlations across taxonomic levels and core microbiomes resolved to taxonomic levels. Correlations provided to order level and treatment type (primary or secondary).

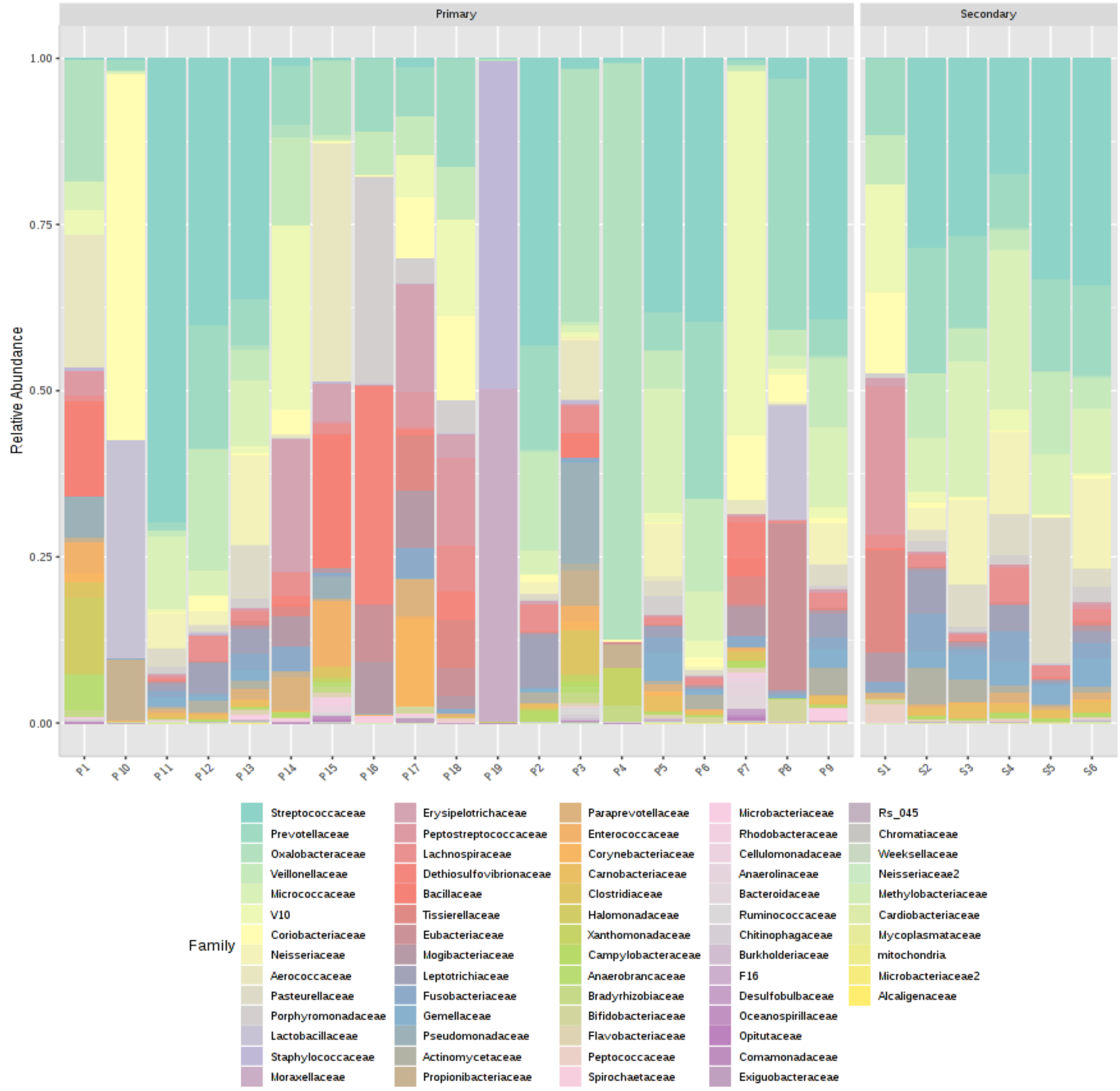

Supplementary Figure S3. Correlations across taxonomic levels and core microbiomes resolved to taxonomic levels. Correlations provided to family level and treatment type (primary or secondary).

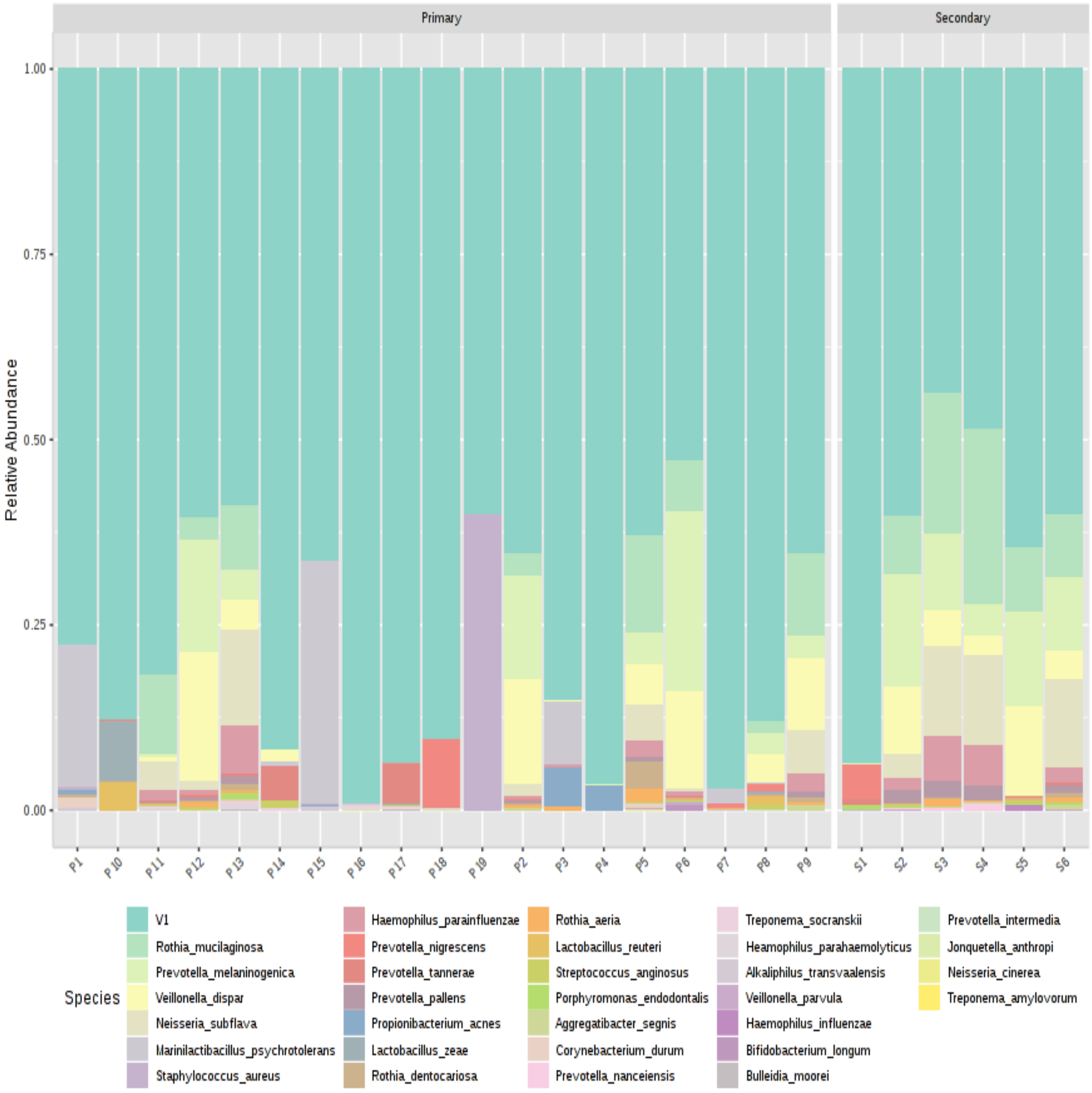

Supplementary Figure S4. Correlations across taxonomic levels and core microbiomes resolved to taxonomic levels. Correlations provided to species level and treatment type (primary or secondary).

Supplementary Table S1. OTUs identified in this study

#TAXONOMY	Kingdom	Phylum	Class	Order	Family	Genus	Species
OTU002	Bacteria	*Actinobacteria*	*Actinobacteria*	*Actinomycetales*	*Actinomycetaceae*		
OTU003	Bacteria	*Actinobacteria*	*Actinobacteria*	*Actinomycetales*	*Actinomycetaceae*	*Actinomyces*	
OTU004	Bacteria	*Actinobacteria*	*Actinobacteria*	*Actinomycetales*	*Cellulomonadaceae*	*Actinotalea*	
OTU005	Bacteria	*Actinobacteria*	*Actinobacteria*	*Actinomycetales*	*Corynebacteriaceae*	*Corynebacterium*	
OTU006	Bacteria	*Actinobacteria*	*Actinobacteria*	*Actinomycetales*	*Corynebacteriaceae*	*Corynebacterium*	*Corynebacterium durum*
OTU007	Bacteria	*Actinobacteria*	*Actinobacteria*	*Actinomycetales*	*Microbacteriaceae*		
OTU008	Bacteria	*Actinobacteria*	*Actinobacteria*	*Actinomycetales*	*Microbacteriaceae2*		
OTU009	Bacteria	*Actinobacteria*	*Actinobacteria*	*Actinomycetales*	*Micrococcaceae*	*Nesterenkonia*	
OTU010	Bacteria	*Actinobacteria*	*Actinobacteria*	*Actinomycetales*	*Micrococcaceae*	*Rothia*	
OTU011	Bacteria	*Actinobacteria*	*Actinobacteria*	*Actinomycetales*	*Micrococcaceae*	*Rothia*	*Rothia aeria*
OTU012	Bacteria	*Actinobacteria*	*Actinobacteria*	*Actinomycetales*	*Micrococcaceae*	*Rothia*	*Rothia dentocariosa*
OTU013	Bacteria	*Actinobacteria*	*Actinobacteria*	*Actinomycetales*	*Micrococcaceae*	*Rothia*	*Rothia mucilaginosa*
OTU014	Bacteria	*Actinobacteria*	*Actinobacteria*	*Actinomycetales*	*Propionibacteriaceae*		
OTU015	Bacteria	*Actinobacteria*	*Actinobacteria*	*Actinomycetales*	*Propionibacteriaceae*	*Propionibacterium*	*Propionibacterium acnes*
OTU016	Bacteria	*Actinobacteria*	*Actinobacteria*	*Bifidobacteriales*	*Bifidobacteriaceae*		
OTU017	Bacteria	*Actinobacteria*	*Actinobacteria*	*Bifidobacteriales*	*Bifidobacteriaceae*	*Bifidobacterium*	
OTU018	Bacteria	*Actinobacteria*	*Actinobacteria*	*Bifidobacteriales*	*Bifidobacteriaceae*	*Bifidobacterium*	*Bifidobacterium longum*
OTU019	Bacteria	*Actinobacteria*	*Actinobacteria*	*Bifidobacteriales*	*Bifidobacteriaceae*	*Scardovia*	
OTU020	Bacteria	*Actinobacteria*	*Coriobacteriia*	*Coriobacteriales*	*Coriobacteriaceae*		
OTU021	Bacteria	*Actinobacteria*	*Coriobacteriia*	*Coriobacteriales*	*Coriobacteriaceae*	*Atopobium*	
OTU022	Bacteria	*Actinobacteria*	*Coriobacteriia*	*Coriobacteriales*	*Coriobacteriaceae*	*Slackia*	
OTU023	Bacteria	*Bacteroidetes*	*Bacteroidia*	*Bacteroidales*			
OTU024	Bacteria	*Bacteroidetes*	*Bacteroidia*	*Bacteroidales*	*Bacteroidaceae*	*Bacteroides*	
OTU025	Bacteria	*Bacteroidetes*	*Bacteroidia*	*Bacteroidales*	*Bacteroidaceae*	*Bacteroides*	*Bacteroides uniformis*

Table S1. *Cont.*

OTU026	Bacteria	*Bacteroidetes*	*Bacteroidia*	*Bacteroidales*	*Porphyromonadaceae*	*Dysgonomonas*	
OTU027	Bacteria	*Bacteroidetes*	*Bacteroidia*	*Bacteroidales*	*Porphyromonadaceae*	*Paludibacter*	
OTU028	Bacteria	*Bacteroidetes*	*Bacteroidia*	*Bacteroidales*	*Porphyromonadaceae*	*Porphyromonas*	
OTU029	Bacteria	*Bacteroidetes*	*Bacteroidia*	*Bacteroidales*	*Porphyromonadaceae*	*Porphyromonas*	*Porphyromonas endodontalis*
OTU030	Bacteria	*Bacteroidetes*	*Bacteroidia*	*Bacteroidales*	*Porphyromonadaceae*	*Tannerella*	
OTU031	Bacteria	*Bacteroidetes*	*Bacteroidia*	*Bacteroidales*	*Prevotellaceae*	*Prevotella*	
OTU032	Bacteria	*Bacteroidetes*	*Bacteroidia*	*Bacteroidales*	*Prevotellaceae*	*Prevotella*	*Prevotella intermedia*
OTU033	Bacteria	*Bacteroidetes*	*Bacteroidia*	*Bacteroidales*	*Prevotellaceae*	*Prevotella*	*Prevotella melaninogenica*
OTU034	Bacteria	*Bacteroidetes*	*Bacteroidia*	*Bacteroidales*	*Prevotellaceae*	*Prevotella*	*Prevotella nanceiensis*
OTU035	Bacteria	*Bacteroidetes*	*Bacteroidia*	*Bacteroidales*	*Prevotellaceae*	*Prevotella*	*Prevotella nigrescens*
OTU036	Bacteria	*Bacteroidetes*	*Bacteroidia*	*Bacteroidales*	*Prevotellaceae*	*Prevotella*	*Prevotella pallens*
OTU037	Bacteria	*Bacteroidetes*	*Bacteroidia*	*Bacteroidales*	*[Paraprevotellaceae]*	*[Prevotella]*	
OTU038	Bacteria	*Bacteroidetes*	*Bacteroidia*	*Bacteroidales*	*[Paraprevotellaceae]*	*[Prevotella]*	*[Prevotella] tannerae*
OTU039	Bacteria	*Bacteroidetes*	*Flavobacteriia*	*Flavobacteriales*	*Flavobacteriaceae*	*Capnocytophaga*	
OTU040	Bacteria	*Bacteroidetes*	*Flavobacteriia*	*Flavobacteriales*	*Flavobacteriaceae*	*Flavobacterium*	
OTU041	Bacteria	*Bacteroidetes*	*Flavobacteriia*	*Flavobacteriales*	*[Weeksellaceae]*		
OTU042	Bacteria	*Bacteroidetes*	*[Saprospirae]*	*[Saprospirales]*	*Chitinophagaceae*	*Sediminibacterium*	
OTU043	Bacteria	*Chloroflexi*	*Anaerolineae*	*Anaerolineales*	*Anaerolinaceae*	*SHD-231*	
OTU044	Bacteria	*Cyanobacteria*	*Chloroplast*	*Streptophyta*			
OTU045	Bacteria	*Firmicutes*	*Bacilli*				
OTU046	Bacteria	*Firmicutes*	*Bacilli*	*Bacillales*			
OTU047	Bacteria	*Firmicutes*	*Bacilli*	*Bacillales*	*Bacillaceae*		
OTU048	Bacteria	*Firmicutes*	*Bacilli*	*Bacillales*	*Bacillaceae*		
OTU049	Bacteria	*Firmicutes*	*Bacilli*	*Bacillales*	*Bacillaceae*	*Anaerobacillus*	
OTU050	Bacteria	*Firmicutes*	*Bacilli*	*Bacillales*	*Staphylococcaceae*	*Staphylococcus*	

Table S1. *Cont.*

OTU051	Bacteria	*Firmicutes*	*Bacilli*	*Bacillales*	*Staphylococcaceae*	*Staphylococcus*	*Staphylococcus aureus*
OTU052	Bacteria	*Firmicutes*	*Bacilli*	*Bacillales*	*[Exiguobacteraceae]*	*Exiguobacterium*	
OTU053	Bacteria	*Firmicutes*	*Bacilli*	*Gemellales*	*Gemellaceae*		
OTU054	Bacteria	*Firmicutes*	*Bacilli*	*Gemellales*	*Gemellaceae*	*Gemella*	
OTU055	Bacteria	*Firmicutes*	*Bacilli*	*Lactobacillales*			
OTU056	Bacteria	*Firmicutes*	*Bacilli*	*Lactobacillales*	*Aerococcaceae*		
OTU057	Bacteria	*Firmicutes*	*Bacilli*	*Lactobacillales*	*Aerococcaceae*	*Alkalibacterium*	
OTU058	Bacteria	*Firmicutes*	*Bacilli*	*Lactobacillales*	*Aerococcaceae*	*Alloiococcus*	
OTU059	Bacteria	*Firmicutes*	*Bacilli*	*Lactobacillales*	*Aerococcaceae*	*Marinilactibacillus*	*Marinilactibacillus psychrotolerans*
OTU060	Bacteria	*Firmicutes*	*Bacilli*	*Lactobacillales*	*Carnobacteriaceae*	*Granulicatella*	
OTU061	Bacteria	*Firmicutes*	*Bacilli*	*Lactobacillales*	*Enterococcaceae*		
OTU062	Bacteria	*Firmicutes*	*Bacilli*	*Lactobacillales*	*Enterococcaceae*	*Enterococcus*	
OTU063	Bacteria	*Firmicutes*	*Bacilli*	*Lactobacillales*	*Enterococcaceae*	*Vagococcus*	
OTU064	Bacteria	*Firmicutes*	*Bacilli*	*Lactobacillales*	*Lactobacillaceae*		
OTU065	Bacteria	*Firmicutes*	*Bacilli*	*Lactobacillales*	*Lactobacillaceae*	*Lactobacillus*	
OTU066	Bacteria	*Firmicutes*	*Bacilli*	*Lactobacillales*	*Lactobacillaceae*	*Lactobacillus*	*Lactobacillus reuteri*
OTU067	Bacteria	*Firmicutes*	*Bacilli*	*Lactobacillales*	*Lactobacillaceae*	*Lactobacillus*	*Lactobacillus zeae*
OTU068	Bacteria	*Firmicutes*	*Bacilli*	*Lactobacillales*	*Streptococcaceae*	*Streptococcus*	
OTU069	Bacteria	*Firmicutes*	*Bacilli*	*Lactobacillales*	*Streptococcaceae*	*Streptococcus*	*Streptococcus anginosus*
OTU070	Bacteria	*Firmicutes*	*Bacilli*	*Lactobacillales*	*Streptococcaceae*	*Streptococcus*	*Streptococcus sobrinus*
OTU071	Bacteria	*Firmicutes*	*Clostridia*	*Clostridiales2*			
OTU072	Bacteria	*Firmicutes*	*Clostridia*	*Clostridiales*			
OTU073	Bacteria	*Firmicutes*	*Clostridia*	*Clostridiales*	*Clostridiaceae*		
OTU074	Bacteria	*Firmicutes*	*Clostridia*	*Clostridiales*	*Clostridiaceae*	*Alkaliphilus*	*Alkaliphilus transvaalensis*

Table S1. *Cont.*

OTU075	Bacteria	*Firmicutes*	*Clostridia*	*Clostridiales*	*Clostridiaceae*	*Geosporobacter_Thermotalea*	
OTU076	Bacteria	*Firmicutes*	*Clostridia*	*Clostridiales*	*Eubacteriaceae*	*Pseudoramibacter_Eubacterium*	
OTU077	Bacteria	*Firmicutes*	*Clostridia*	*Clostridiales*	*Lachnospiraceae*		
OTU078	Bacteria	*Firmicutes*	*Clostridia*	*Clostridiales*	*Lachnospiraceae*	*Butyrivibrio*	
OTU079	Bacteria	*Firmicutes*	*Clostridia*	*Clostridiales*	*Lachnospiraceae*	*Catonella*	
OTU080	Bacteria	*Firmicutes*	*Clostridia*	*Clostridiales*	*Lachnospiraceae*	*Moryella1*	
OTU081	Bacteria	*Firmicutes*	*Clostridia*	*Clostridiales*	*Lachnospiraceae*	*Moryella2*	
OTU082	Bacteria	*Firmicutes*	*Clostridia*	*Clostridiales*	*Lachnospiraceae*	*Oribacterium*	
OTU083	Bacteria	*Firmicutes*	*Clostridia*	*Clostridiales*	*Peptococcaceae*	*Peptococcus*	
OTU084	Bacteria	*Firmicutes*	*Clostridia*	*Clostridiales*	*Peptostreptococcaceae*		
OTU085	Bacteria	*Firmicutes*	*Clostridia*	*Clostridiales*	*Peptostreptococcaceae*	*Filifactor*	
OTU086	Bacteria	*Firmicutes*	*Clostridia*	*Clostridiales*	*Peptostreptococcaceae*	*Peptostreptococcus*	
OTU087	Bacteria	*Firmicutes*	*Clostridia*	*Clostridiales*	*Ruminococcaceae*	*Ethanoligenens*	
OTU088	Bacteria	*Firmicutes*	*Clostridia*	*Clostridiales*	*Veillonellaceae*		
OTU089	Bacteria	*Firmicutes*	*Clostridia*	*Clostridiales*	*Veillonellaceae*	*Dialister*	
OTU090	Bacteria	*Firmicutes*	*Clostridia*	*Clostridiales*	*Veillonellaceae*	*Megasphaera*	
OTU091	Bacteria	*Firmicutes*	*Clostridia*	*Clostridiales*	*Veillonellaceae*	*Schwartzia*	
OTU092	Bacteria	*Firmicutes*	*Clostridia*	*Clostridiales*	*Veillonellaceae*	*Selenomonas*	
OTU093	Bacteria	*Firmicutes*	*Clostridia*	*Clostridiales*	*Veillonellaceae*	*Veillonella2*	
OTU094	Bacteria	*Firmicutes*	*Clostridia*	*Clostridiales*	*Veillonellaceae*	*Veillonella*	*Veillonella dispar*
OTU095	Bacteria	*Firmicutes*	*Clostridia*	*Clostridiales*	*Veillonellaceae*	*Veillonella*	*Veillonella parvula*
OTU096	Bacteria	*Firmicutes*	*Clostridia*	*Clostridiales*	*[Mogibacteriaceae]*		
OTU097	Bacteria	*Firmicutes*	*Clostridia*	*Clostridiales*	*[Mogibacteriaceae]*	*Anaerovorax*	
OTU098	Bacteria	*Firmicutes*	*Clostridia*	*Clostridiales*	*[Mogibacteriaceae]*	*Mogibacterium*	
OTU099	Bacteria	*Firmicutes*	*Clostridia*	*Clostridiales*	*[Tissierellaceae]*		

Table S1. *Cont.*

OTU100	Bacteria	*Firmicutes*	*Clostridia*	*Clostridiales*	*[Tissierellaceae]*	*Parvimonas*	
OTU101	Bacteria	*Firmicutes*	*Clostridia*	*Clostridiales*	*[Tissierellaceae]*	*Peptoniphilus*	
OTU102	Bacteria	*Firmicutes*	*Clostridia*	*Natranaerobiales*	*Anaerobrancaceae*		
OTU103	Bacteria	*Firmicutes*	*Erysipelotrichi*	*Erysipelotrichales*	*Erysipelotrichaceae*	*Bulleidia*	
OTU104	Bacteria	*Firmicutes*	*Erysipelotrichi*	*Erysipelotrichales*	*Erysipelotrichaceae*	*Bulleidia*	*Bulleidia moorei*
OTU105	Bacteria	*Firmicutes*	*Erysipelotrichi*	*Erysipelotrichales*	*Erysipelotrichaceae*	*PSB-M-3*	
OTU106	Bacteria	*Firmicutes*	*Erysipelotrichi*	*Erysipelotrichales*	*Erysipelotrichaceae*	*[Eubacterium]*	*[Eubacterium] dolichum*
OTU107	Bacteria	*Fusobacteria*	*Fusobacteriia*	*Fusobacteriales*	*Fusobacteriaceae*	*Fusobacterium*	
OTU108	Bacteria	*Fusobacteria*	*Fusobacteriia*	*Fusobacteriales*	*Leptotrichiaceae*		
OTU109	Bacteria	*Fusobacteria*	*Fusobacteriia*	*Fusobacteriales*	*Leptotrichiaceae*	*Leptotrichia*	
OTU110	Bacteria	*Proteobacteria*	*Alphaproteobacteria*	*Rhizobiales*	*Bradyrhizobiaceae*		
OTU111	Bacteria	*Proteobacteria*	*Alphaproteobacteria*	*Rhizobiales*	*Methylobacteriaceae*	*Methylobacterium*	
OTU112	Bacteria	*Proteobacteria*	*Alphaproteobacteria*	*Rhodobacterales*	*Rhodobacteraceae*		
OTU113	Bacteria	*Proteobacteria*	*Alphaproteobacteria*	*Rhodobacterales*	*Rhodobacteraceae*	*Paracoccus*	
OTU114	Bacteria	*Proteobacteria*	*Alphaproteobacteria*	*Rickettsiales*	*mitochondria*		
OTU115	Bacteria	*Proteobacteria*	*Betaproteobacteria*	*Burkholderiales*	*Alcaligenaceae*	*Sutterella*	
OTU116	Bacteria	*Proteobacteria*	*Betaproteobacteria*	*Burkholderiales*	*Burkholderiaceae*	*Lautropia*	
OTU117	Bacteria	*Proteobacteria*	*Betaproteobacteria*	*Burkholderiales*	*Comamonadaceae*		
OTU118	Bacteria	*Proteobacteria*	*Betaproteobacteria*	*Burkholderiales*	*Oxalobacteraceae*		
OTU119	Bacteria	*Proteobacteria*	*Betaproteobacteria*	*Neisseriales*	*Neisseriaceae2*		
OTU120	Bacteria	*Proteobacteria*	*Betaproteobacteria*	*Neisseriales*	*Neisseriaceae*		
OTU121	Bacteria	*Proteobacteria*	*Betaproteobacteria*	*Neisseriales*	*Neisseriaceae*	*Kingella*	
OTU122	Bacteria	*Proteobacteria*	*Betaproteobacteria*	*Neisseriales*	*Neisseriaceae*	*Neisseria*	
OTU123	Bacteria	*Proteobacteria*	*Betaproteobacteria*	*Neisseriales*	*Neisseriaceae*	*Neisseria2*	
OTU124	Bacteria	*Proteobacteria*	*Betaproteobacteria*	*Neisseriales*	*Neisseriaceae*	*Neisseria*	*Neisseria cinerea*

Table S1. *Cont.*

OTU125	Bacteria	*Proteobacteria*	*Betaproteobacteria*	*Neisseriales*	*Neisseriaceae*	*Neisseria*	*Neisseria subflava*
OTU126	Bacteria	*Proteobacteria*	*Deltaproteobacteria*	*Desulfobacterales*	*Desulfobulbaceae*	*Desulfobulbus*	
OTU127	Bacteria	*Proteobacteria*	*Deltaproteobacteria*	*Desulfovibrionales*	*Desulfovibrionaceae*	*Bilophila*	
OTU128	Bacteria	*Proteobacteria*	*Deltaproteobacteria*	*Desulfovibrionales*	*Desulfovibrionaceae*	*Desulfovibrio*	
OTU129	Bacteria	*Proteobacteria*	*Epsilonproteobacteria*	*Campylobacterales*	*Campylobacteraceae*	*Campylobacter*	
OTU130	Bacteria	*Proteobacteria*	*Gammaproteobacteria*	*Alteromonadales*	*[Chromatiaceae]*	*Alkalimonas*	
OTU131	Bacteria	*Proteobacteria*	*Gammaproteobacteria*	*Cardiobacteriales*	*Cardiobacteriaceae*	*Cardiobacterium*	
OTU132	Bacteria	*Proteobacteria*	*Gammaproteobacteria*	*Oceanospirillales*	*Halomonadaceae*	*Halomonas*	
OTU133	Bacteria	*Proteobacteria*	*Gammaproteobacteria*	*Oceanospirillales*	*Oceanospirillaceae*	*Nitrincola*	
OTU134	Bacteria	*Proteobacteria*	*Gammaproteobacteria*	*Pasteurellales*	*Pasteurellaceae*	*Heamophilus*	*Heamophilus parahaemolyticus*
OTU135	Bacteria	*Proteobacteria*	*Gammaproteobacteria*	*Pasteurellales*	*Pasteurellaceae*	*Aggregatibacter*	*Aggregatibacter segnis*
OTU136	Bacteria	*Proteobacteria*	*Gammaproteobacteria*	*Pasteurellales*	*Pasteurellaceae*	*Haemophilus*	
OTU137	Bacteria	*Proteobacteria*	*Gammaproteobacteria*	*Pasteurellales*	*Pasteurellaceae*	*Haemophilus2*	
OTU138	Bacteria	*Proteobacteria*	*Gammaproteobacteria*	*Pasteurellales*	*Pasteurellaceae*	*Haemophilus*	*Haemophilus influenzae*
OTU139	Bacteria	*Proteobacteria*	*Gammaproteobacteria*	*Pasteurellales*	*Pasteurellaceae*	*Haemophilus*	*Haemophilus parainfluenzae*
OTU140	Bacteria	*Proteobacteria*	*Gammaproteobacteria*	*Pseudomonadales*	*Moraxellaceae*	*Moraxella*	
OTU141	Bacteria	*Proteobacteria*	*Gammaproteobacteria*	*Pseudomonadales*	*Pseudomonadaceae*	*Pseudomonas*	
OTU142	Bacteria	*Proteobacteria*	*Gammaproteobacteria*	*Xanthomonadales*	*Xanthomonadaceae*		
OTU143	Bacteria	*SR1*					
OTU144	Bacteria	*Spirochaetes*	*Spirochaetes*	*Spirochaetales*	*Spirochaetaceae*	*Treponema*	
OTU145	Bacteria	*Spirochaetes*	*Spirochaetes*	*Spirochaetales*	*Spirochaetaceae*	*Treponema*	*Treponema amylovorum*
OTU146	Bacteria	*Spirochaetes*	*Spirochaetes*	*Spirochaetales*	*Spirochaetaceae*	*Treponema*	*Treponema socranskii*
OTU147	Bacteria	*Synergistetes*	*Synergistia*	*Synergistales*	*Dethiosulfovibrionaceae*		
OTU148	Bacteria	*Synergistetes*	*Synergistia*	*Synergistales*	*Dethiosulfovibrionaceae*	*Jonquetella*	*Jonquetella anthropi*

Table S1. *Cont.*

OTU149	Bacteria	*Synergistetes*	*Synergistia*	*Synergistales*	*Dethiosulfovibrionaceae*	*TG5*
OTU150	Bacteria	TM7	TM7-3			
OTU151	Bacteria	TM7	TM7-3	CW040		
OTU152	Bacteria	TM7	TM7-3	CW040	F16	
OTU153	Bacteria	TM7	TM7-3	I025	Rs-045	
OTU154	Bacteria	*Tenericutes*	*Mollicutes*	*Mycoplasmatales*	*Mycoplasmataceae*	*Mycoplasma*
OTU155	Bacteria	*Verrucomicrobia*	*Opitutae*	*Opitutales*	*Opitutaceae*	*Opitutus*

Journal of
Clinical Medicine

MDPI
